# Supplementary material for: ATP Production Relies on Fatty Acid Oxidation Rather than Glycolysis in Pancreatic Ductal Adenocarcinoma
Source: Cancers (Basel). 2020 Sep 1;12(9):2477. doi: 10.3390/cancers12092477 (PMC7564784; doi:10.3390/cancers12092477)
Supplement: Supplementary file 1 [file cancers-12-02477-s001.pdf]

cancers

Supplementary Material

ATP Production Relies on Fatty Acid Oxidation Rather than Glycolysis in Pancreatic Ductal Adenocarcinoma

Jae-Seon Lee, Su-Jin Oh, Hyun-Jung Choi, Joon Hee Kang, Seon-Hyeong Lee, Ji Sun Ha, Sang Myung Woo, Hyonchol Jang, Ho Lee, and Soo-Youl Kim

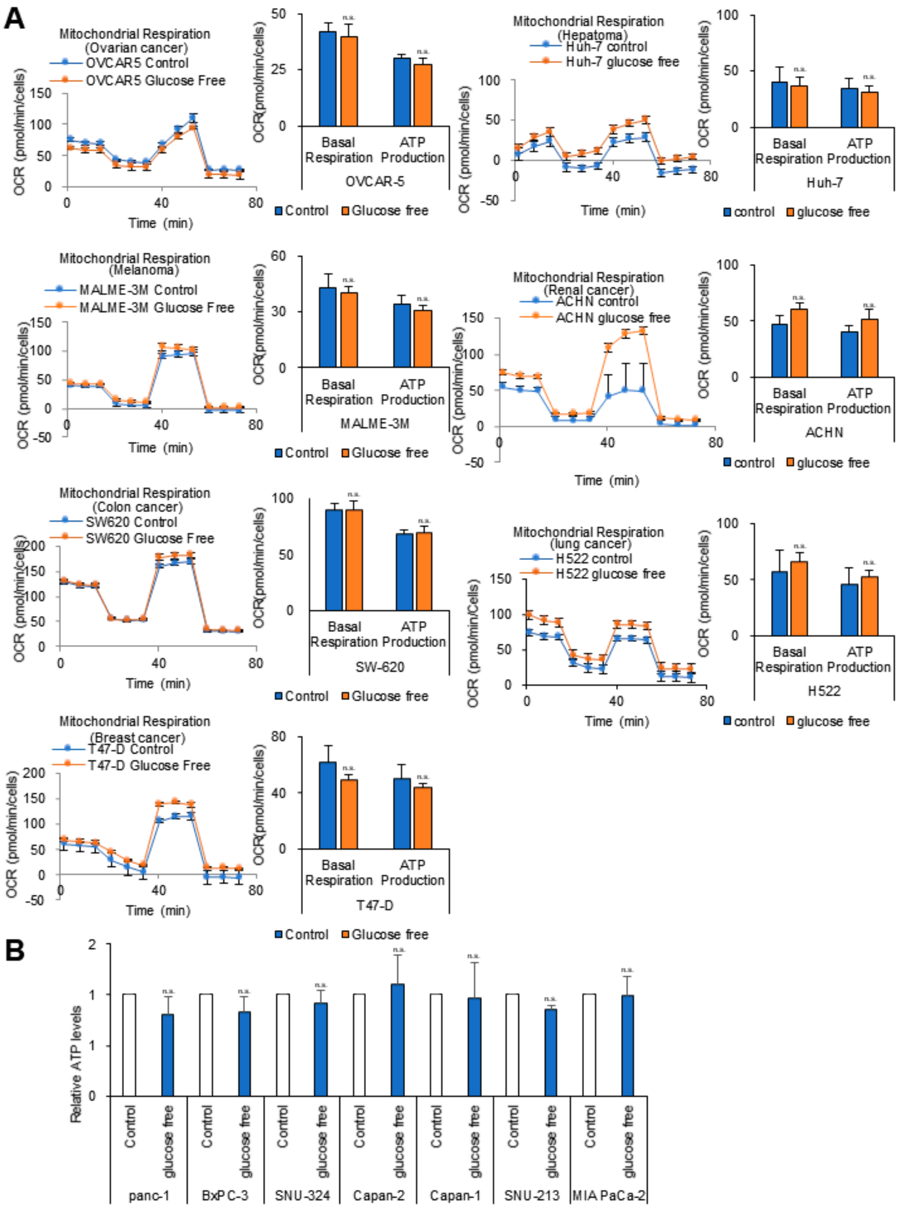
A
Mitochondrial Respiration (Ovarian cancer)
OVCAR5 Control
OVCAR5 Glucose Free
OCR (pmol/min/cells)
Time (min)
Basal Respiration
ATP Production
OVCAR-5
Control
Glucose free
n.s.
Mitochondrial Respiration (Hepatoma)
Huh-7 control
Huh-7 glucose free
Huh-7
control
glucose free
Mitochondrial Respiration (Melanoma)
MALME-3M Control
MALME-3M Glucose Free
MALME-3M
Mitochondrial Respiration (Renal cancer)
ACHN control
ACHN glucose free
ACHN
Mitochondrial Respiration (Colon cancer)
SW620 Control
SW620 Glucose Free
SW-620
Mitochondrial Respiration (lung cancer)
H522 control
H522 glucose free
OCR (pmol/min/Cells)
H522
Mitochondrial Respiration (Breast cancer)
T47-D Control
T47-D Glucose Free
T47-D
B
Relative ATP levels
Control
glucose free
panc-1
BxPC-3
SNU-324
Capan-2
Capan-1
SNU-213
MIA PaCa-2

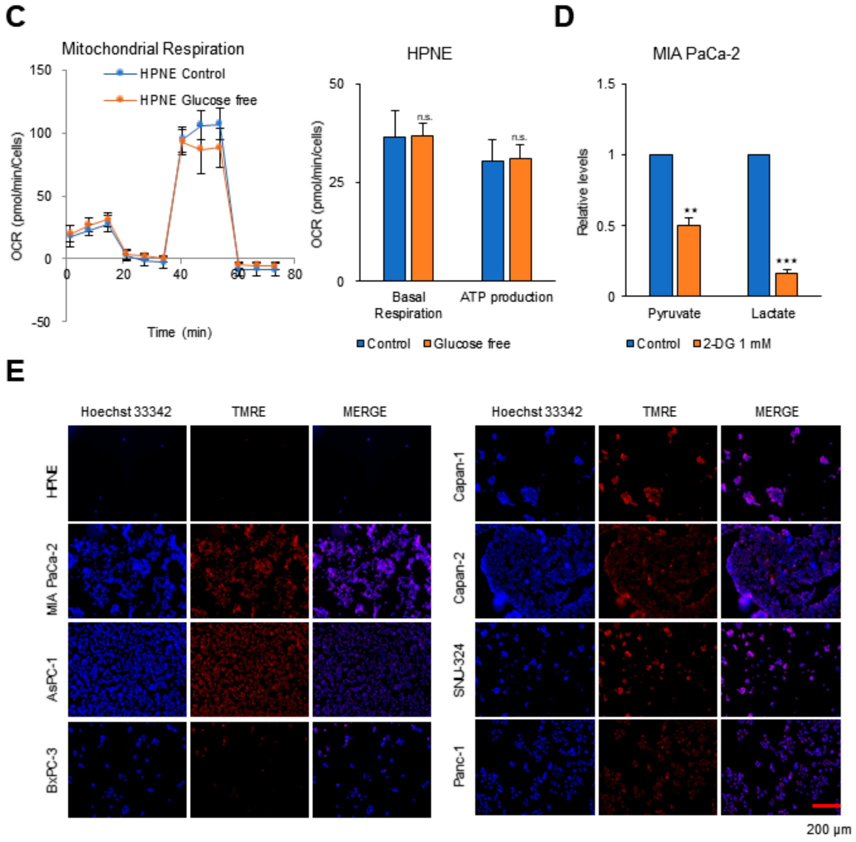

Figure S1. Glucose deprivation was not able to affect basal consumption rate and ATP production. (**A**) Seahorse XF analysis of various other cancer cells (Ovarian, Melanoma, Colon, Breast, Hepatoma, Renal, Lung cancer cells) treated sequentially with oligomycin, the chemical uncoupler FCCP, and antimycin A/ Rotenone. The oxygen consumption rate (OCR) was analysed using the Seahorse XFe analyzer in normal condition media compared to glucose deprivation media. (**B**) ATP levels were measured using an ATP Colorimetric/Fluorometric Assay kit after PDAC cells were incubated with glucose free media for 24 h. (**C**) Seahorse XF analysis of HPNE cells treated sequentially with oligomycin, the chemical uncoupler FCCP and antimycin A/Rotenone. The oxygen consumption rate was determined using the Seahorse XF96 analyzer in normal medium compared with glucose deprivation medium. (**D**) Pyruvate and lactate were measured by targeted LC-MS/MS after MIA PaCa-2 cells were treated with 2-DG 1 mM for 24 h. (**E**) Mitochondrial membrane potential of pancreatic cancer cell lines and HPNE was determined by TMRE staining and live cell imaging. Scale bar = 200 µm. n.s. (not significant), * $p < 0.05$, ** $p < 0.01$, *** $p < 0.001$.

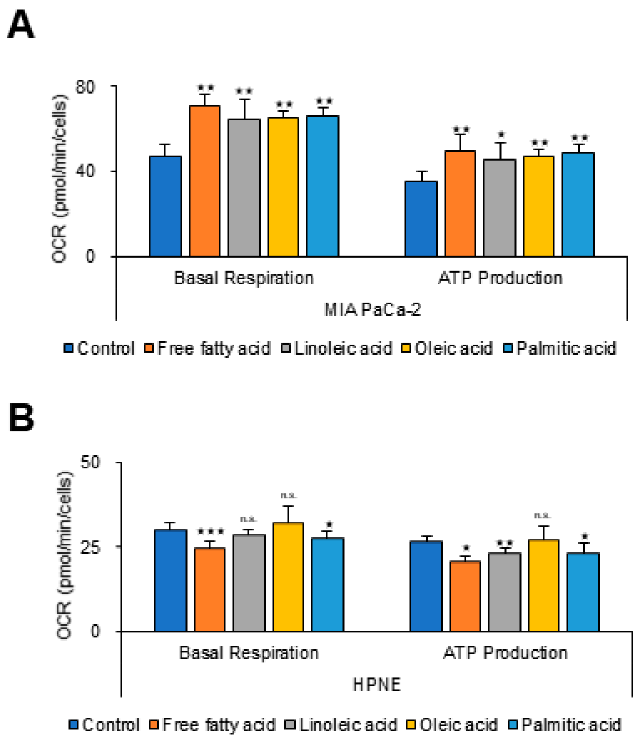

Figure S2. Effect of supplementary fatty acids on basal respiration and ATP production in PDAC and normal cells. (**A** and **B**) To determine whether fatty acids such as linoleic acid (grey), oleic acid (yellow), and palmitic acid (light blue) induce ATP production thorough FAO, (**A**) PDAC cells and (**B**) normal HPNE cells were treated with supplementary fatty acids under normal culture conditions. The oxygen consumption rate (OCR) was analysed using the Seahorse XFe analyzer. Basal respiration and ATP Production levels in MIA PaCa-2 and HPNE Cells for supplementary fatty acids. n.s. (not significant), * $p < 0.05$, ** $p < 0.01$, *** $p < 0.001$.

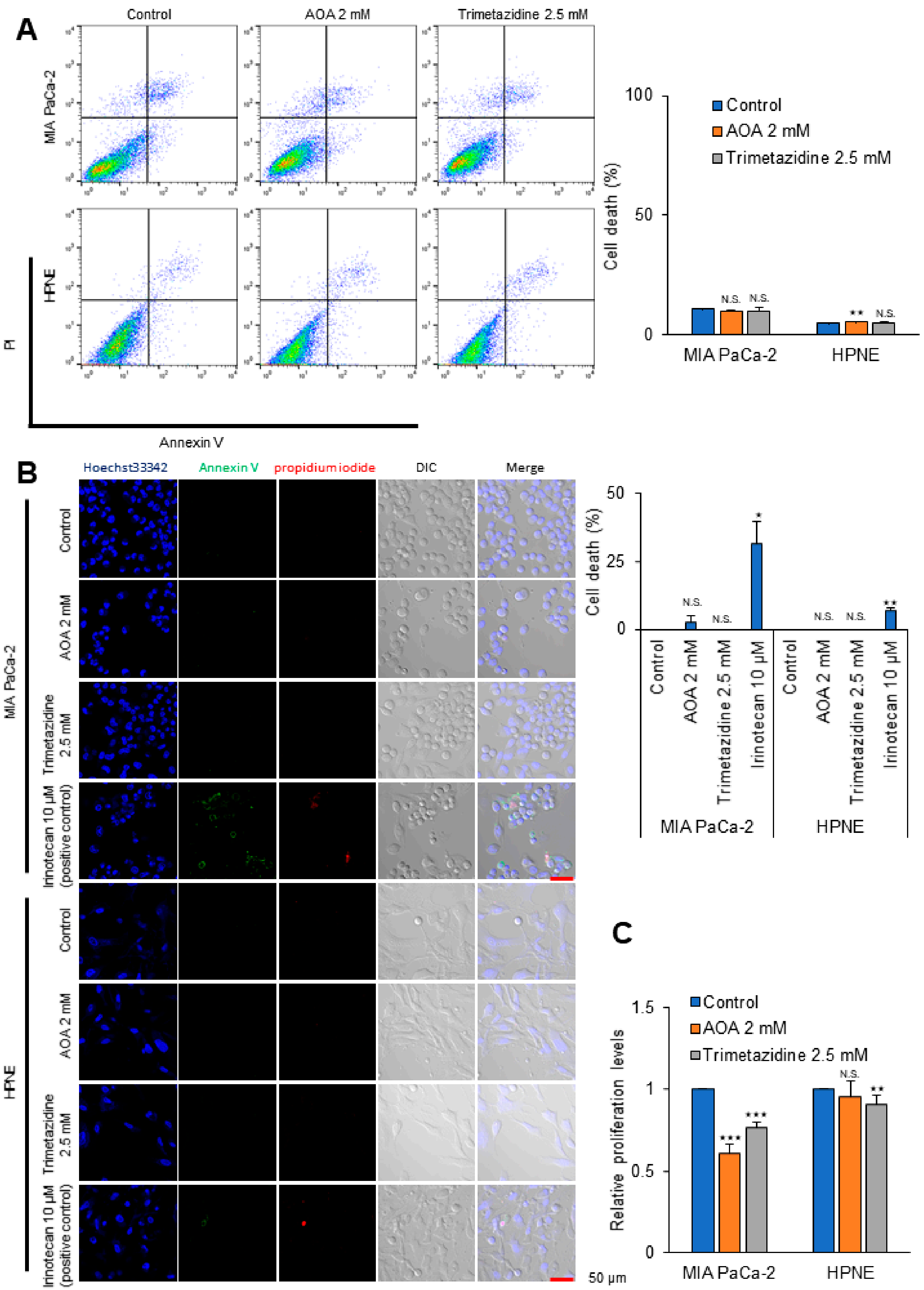

Figure S3. Effect of inhibition of MAS and FAO on cell proliferation and cell death. (**A** and **B**) Cell death analysis using annexin V cell death detection kit was performed with MIA PaCa-2 and normal HPNE cells after inhibitors treatment of aminooxyacetic acid (AOA; malate-aspartate shuttle inhibitor) or trimetazidine (fatty acid oxidation inhibitor) or irinotecan (positive control for cell death) for 24 h. (**A**) Cell death was analyzed by the Flow cytometry and (**B**) confocal microscope. Cell death

was not observed by treatment of AOA or trimetazidine both in MIA PaCa-2 and HPNE while it was observed by treatment of irinotecan. (**C**) Cell proliferation was measured using SRB assay after MIA PaCa-2 and HPNE cells were treated with AOA or trimetazidine for 24 h. Treatment of AOA and trimetazidine down regulated cell proliferation of MIA PaCa-2 but did not reduce the growth of HPNE. Scale bar = 50 μm. N.S. (not significant), * $p < 0.05$, ** $p < 0.01$, *** $p < 0.001$.

Table S1. Formula of High-Fat Diet and Low-Fat Diet.

	**HFD**		**HFD Control (High Fat Diet)**		**LFD Control (Low Fat Diet)**		**LFD**	
	gm%	kcal%	gm%	kcal%	gm%	kcal%	gm%	kcal%
Protein	26.2	20	19.2	20	20.3	20.8	20.3	22.2
Carbohydrate	26.3	20	67.3	70	66.0	67.7	71.0	77.8
Fat	34.9	60	4.3	10	5.0	11.5	0.0	0.0
Total	-	100	-	100	-	100.0	-	100.0
Kcal/gm	5.24	-	3.85	-	3.90	-	3.65	-
Ingredient	gm	kcal	gm	kcal	gm	kcal	gm	kcal
Casein, 30 Mesh	200	800	200	800	200	800	200	800
L-Cystine	3	12	3	12	-	-	-	-
DL-Methionine	-	-	-	-	3	12	3	12
Corn Starch	0	0	315	1260	150	600	0	0
Sucrose	68.8	275.2	350	1400	500	2000	700	2800
Maltodextrin 10	125	500	35	140	-	-	-	-
Cellulose, BW200	50	0	50	0	50	0	50	0
Corn oil	-	-	-	-	50	450	0	0
Soybean Oil	25	225	25	225	-	-	-	-
Lard	245	2205	20	180	-	-	-	-
Mineral Mix S10001	10	0	10	0	35	0	35	0
Vitamin Mix V10001	10	40	10	40	10	40	10	40
Choline Bitartrate	2	0	2	0	2	0	2	0
Dicalcium Phosphate	13	0	13	0	-	-	-	-
Calcium Carbonate	5.5	0	5.5	0	-	-	-	-
Potassium Citrate, 1H2O	16.5	0	16.5	0	-	-	-	-
FD&C Blue Dye #1	0.05	0	-	-	-	-	-	-
FD&C Yellow Dye #5	-	-	0.05	0	-	-	-	-
Total	773.85	4057	1055.05	4057	1000	3902	1000	3652

*HFD: High Fat Diet; LFD: Low Fat Diet

Table S2. Alterations of genes in pancreatic cancer cell lines.

**Cell line**	**Abnormalities of p16 gene**	**Abnormalities of p53 gene**	**Abnormalities of K-ras gene**

	Codon	a.a change	Codon	a.a change	Codon	a.a change
SNU-213	wt		175	Arg - His	12	Gly - Val
SNU-324	22	Ala - Val	wt		wt	
MIA PaCa-2	null		248	Arg - Trp	12	Gly - Cys
Panc-1	null		273	Arg - His	12	Gly - Asp
BxPC-3	null		220	Tyr-Cys	wt	
Capan-1	wt		159	Ala - Val	12	Gly - Val
Capan-2	wt		wt		12	Gly - Val
